# Draft Whole-Genome Sequence of *Serratia* sp. Strain TEL, Associated with *Oscheius* sp. TEL-2014 (Nematoda: Rhabditidae) Isolated from a Grassland in South Africa

**DOI:** 10.1128/genomeA.00747-15

**Published:** 2015-07-09

**Authors:** Tiisetso E. Lephoto, Jonathan Featherston, Vincent M. Gray

**Affiliations:** a School of Molecular and Cell Biology, University of the Witwatersrand, Johannesburg, South Africa; b Agricultural Research Council, Biotechnology Platform, Pretoria, South Africa; c School of Molecular and Cell Biology, University of the Witwatersrand, Johannesburg, South Africa

## Abstract

Here, we report on the draft genome sequence of *Serratia* sp. strain TEL, associated with *Oscheius* sp. TEL-2014 (Nematoda: Rhabditidae, KM492926) isolated from a grassland in Suikerbosrand Nature Reserve near Johannesburg in South Africa. *Serratia* sp. strain TEL has a genome size of 5,000,541 bp with 4,647 genes and a G+C content of 59.1%.

## GENOME ANNOUNCEMENT

The majority of entomopathogenic nematode (EPN) species that have been isolated belong to heterorhabditids and steinernematids (1), which act as vectors for insect pathogenic bacteria species belonging to the genera *Photorhabdus* and *Xenorhabdus*, respectively (2). However, other species of nematodes, one of which includes a strain of *Caenorhabditis briggsae*, have also been shown to act as vectors for insect pathogenic strains of *Serratia marcescens* (3).

*Oscheius* nematodes now also act as vectors for insect pathogenic bacteria belonging to the genus *Serratia* (4). Like heterorhabditids and steinernematids, entomopathogenic *Oscheius* species also have a dauer stage in their life cycle (5). During this stage, *Serratia* spp. persist internally within the infective juvenile (IJ) as endosymbionts (6). On infection of an insect host, entomopathogenic *Serratia* is released by the *Oscheius* IJ into the insect’s hemocoel, in an infection process that is very similar to the pathogenic behavior of the heterorhabditids and steinernematids (5). All EPNs complete their cycle by feeding on the bacteria growing inside the host’s hemocoel (7). Once the nutrient reserves have been depleted, nonfeeding *Oscheius* sp. IJs, carrying a colony of endosymbiotic *Serratia* sp. bacteria (8), migrate from the cadaver and survive in a state of anhydrobiosis for extended periods in the soil (9).

In this study, the novel insect pathogenic *Serratia* sp. strain TEL (GenBank accession number KP711410) was isolated from the gut of an IJ of *Oscheius* sp. TEL-2014 (KM492926).

Methods described in reference 10 were employed to isolate *Serratia* sp. strain TEL from *Oscheius* sp. TEL-2014. Whole DNA extraction from solid bacterial colony cultures was done using the ZR bacterial DNA miniprep kit (Zymo Research). Genomic DNA paired-end libraries were generated with the Nextera DNA sample preparation kit (Illumina) and indexed using the Nextera index kit (Illumina). Paired-end (2 × 300 bp) sequencing was performed on a MiSeq Illumina using the MiSeq reagent kit version 3 at the Agricultural Research Council Biotechnology Platform. Quality and adapter trimming was performed with the fastq-mcf toolkit. The genome was assembled using SPADES, and 19 contigs were generated with an average length of 301,767 bp and an *N*_50_ of 200,110 bp. The genome of *Serratia* sp. strain TEL was found to be 5,000,541 bp in size, with a G+C content of 59.1%, which was similar to that of other *Serratia* species (11–13). Furthermore, the contigs were annotated using the NCBI Prokaryotic Genome Automatic Annotation Pipeline.

The annotated features are as follows: 4,647 genes were found, and 4,495 were protein-coding sequences (CDS). The genome contains 36 pseudogenes, 2 CRISPR arrays, 13 rRNA genes with five operons (5S, 16S, 23S), 88 tRNAs, 15 noncoding RNAs, and 9 frameshifted genes.

Several genes involved in virulence, disease, defense, stress response, cell division, motility, and chemotaxis were identified. This draft genome sequence will allow for the investigation of identified genes and will be critical in furthering the understanding of the insect pathogenicity of *Serratia* sp. strain TEL.

### Nucleotide sequence accession numbers. 

This whole-genome shotgun project has been deposited at DDBJ/EMBL/GenBank under the accession number LDEG00000000, which is the first version.
